# Potentiometric Surfactant Sensor with a Pt-Doped Acid-Activated Multi-Walled Carbon Nanotube-Based Ionophore Nanocomposite

**DOI:** 10.3390/s24082388

**Published:** 2024-04-09

**Authors:** Nada Glumac, Milan Momčilović, Iztok Kramberger, Darko Štraus, Nikola Sakač, Elvira Kovač-Andrić, Bojan Đurin, Marija Kraševac Sakač, Kristina Đambić, Marija Jozanović

**Affiliations:** 1 Međimurske Vode D.O.O., 40000 Čakovec, Croatia; nada.glumac@medjimurske-vode.hr; 2 Faculty of Sciences and Mathematics, University of Niš, 18000 Niš, Serbia; milan.momcilovic@pmf.edu.rs; 3 Faculty of Electrical Engineering and Computer Science, University of Maribor, 2000 Maribor, Slovenia; iztok.kramberger@um.si (I.K.); darko.straus@um.si (D.Š.); 4 Faculty of Geotechnical Engineering, University of Zagreb, 42000 Varaždin, Croatia; 5 Department of Chemistry, University of Osijek, 31000 Osijek, Croatia; eakovac@kemija.unios.hr (E.K.-A.); kdjambic@gmail.com (K.Đ.); 6 Department of Civil Engineering, University North, 42000 Varaždin, Croatia; bdjurin@unin.hr; 7 Faculty of Chemical Engineering and Technology, University of Zagreb, 10000 Zagreb, Croatia; mkrasevac@fkit.hr

**Keywords:** potentiometric surfactant sensor, metal-doped MWCNT, surfactants, carbon nanocomposite, sensor

## Abstract

Two new surfactant sensors were developed by synthesizing Pt-doped acid-activated multi-walled carbon nanotubes (Pt@MWCNTs). Two different ionophores using Pt@MWCNTs, a new plasticizer, and (a) cationic surfactant 1,3-dihexadecyl-1H-benzo[d]imidazol-3-ium-DHBI (Pt@MWCNT-DHBI ionophore) and (b) anionic surfactant dodecylbenzenesulfonate-DBS (Pt@MWCNT-DBS ionophore) composites were successfully synthesized and characterized. Both surfactant sensors showed a response to anionic surfactants (dodecylsulfate (SDS) and DBS) and cationic surfactants (cetylpyridinium chloride (CPC) and hexadecyltrimethylammonium bromide (CTAB)). The Pt@MWCNT-DBS sensor showed lower sensitivity than expected with the sub-Nernstian response of ≈23 mV/decade of activity for CPC and CTAB and ≈33 mV/decade of activity for SDS and DBS. The Pt@MWCNT-DHBI surfactant sensor had superior response properties, including a Nernstian response to SDS (59.1 mV/decade) and a near-Nernstian response to DBS (57.5 mV/decade), with linear response regions for both anionic surfactants down to ≈2 × 10^−6^ M. The Pt@MWCNT-DHBI was also useful in critical micellar concentration (CMC) detection. Common anions showed very low interferences with the sensor. The sensor was successfully employed for the potentiometric titration of a technical grade cationic surfactant with good recoveries. The content of cationic surfactants was measured in six samples of complex commercial detergents. The Pt@MWCNT-DHBI surfactant sensor showed good agreement with the ISE surfactant sensor and classical two-phase titration and could be used as an analytical tool in quality control.

## 1. Introduction

Surfactants are organic molecules with the properties of lowering the surface tension of water. Usually, they consist of hydrophilic heads and hydrophobic tails rich in carbohydrates. There are four main types of surfactants: anionic, cationic, amphoteric, and nonionic [1]. They are usually used in everyday life and the industry as detergents for washing, cleaning (anionic), and disinfection (cationic). The global surfactant market is constantly growing. From a medical point of view and life standard, this is positive, but from an environmental and health standpoint, this is a challenge. Surfactants have a negative influence on the environment since they disintegrate cells, prevent oxygen exchange, etc.; they irritate the skin and can cause other health issues. For this reason, it is important to monitor the water constantly and increase quality control during detergent production. Classical methods of surfactant detection include two-phase titration and the MBAS method [2,3]. Both methods are time-consuming, require skilled experts, have a lack of reproducibility, and are in contrast to the principles of green chemistry (since they require toxic organic solvents for extraction). Other more common instrumental methods are chromatography methods like Size-Exclusion Chromatography [4], Ion-Exchange Chromatography [5], HPLC [6], and Gas Chromatography [7]. These methods offer reproducibility but require skilled personnel and are expensive to perform and operate.

To overcome all these issues in surfactant analysis, chemical sensors could be a fast, reliable, and easy-to-use strategy. Surfactant sensors based on liquid-type membranes are the most common. PVC is mixed with the selected plasticizer in a ratio of 1:2 [8]. This composition acts like a solvent for the ionophore (usually 1%). The ionophore is an ion pair usually made of a large cationic surfactant and an oppositely charged lipophilic ion. The most important part of the sensor membrane is the ionophore. Recent advances in liquid membrane-type ion-selective electrodes (ISE) for surfactants offer the use of new molecules for ion-pair synthesis and the use of nanomaterials to improve sensor characteristics [9,10,11]. Nanomaterials can be chemically bound to the ion pair. On the other hand, an inclusion complex with an ion pair or charged surfactant can also act as an ionophore [12,13,14]. An example of the latter is surfactant-selective electrochemical sensors based on single-walled carbon nanotubes (SWCNTs), where CTAB and SDS, adsorbed to the SWCNTs, act as ionophores [15]. In this study, two electrodes were fabricated: CTAB-SWCNT and SWCNT-SDS. The proposed sensors exhibited a Nernstian response of 59.5 mV/decade of activity for SDS and a near-Nernstian response of 57.2 mV/decade of activity for CTAB over a wide concentration range.

The electrochemical characteristics of electrodes depend significantly on their surface properties. The rate of electron transfer can be notably enhanced by the surface functional groups which contain oxygen. Carbon nanotubes (CNTs) exhibit two distinct surface regions due to their specific structures: sidewalls and ends. The defect-free structure of intact nanotubes is similar to the basal planes of pyrolytic graphite. However, cap regions may be potentially more reactive due to a higher curve strain compared to the sidewall. The treatment of carbon nanotubes by physical or chemical means to open their ends generates various oxygen-containing groups, resembling the properties of edge sites on basal planes of pyrolytic graphite [16]. Electrochemical sensors containing multi-walled CNTs have shown superior properties in terms of functional surfaces, small dimensions, great chemical stability, good conductivity, a modifiable sidewall and great mechanical strength, sensitivity, a broad potential window, and low background current [17].

The use of carbon-based nanomaterials in electrochemical sensor development was improved by metal-doped nanomaterials, like metal-doped carbon nanotubes (CNTs) [10,18,19]. This resulted in a conductivity increase, which caused a faster response time and extended operating range [20]. When used in potentiometric sensors, metal-doped nanomaterials offer several advantages as follows: enhanced sensitivity, improved selectivity, a wider detection range, faster response time, improved stability, reduced fouling, and could result in better miniaturization and integration [10,21,22,23,24,25]. The MWCNTs/Fe-Co doped nanocomposite was used for the potentiometric determination of sulpiride in real water samples. Using the metal-doped MWCNTs resulted in many advantages, such as enhanced charge transfer and sensitivity, fast response time, and increased selectivity; the detection limits were 7.6 × 10^−7^ M [21]. The combined presence of carbon nanotubes with their excellent electron-transfer capacity and platinum Pt, which possesses the best catalytic activity among all pure metals and remains the most frequently used catalyst in a variety of applications, can lead to remarkable synergistic effects. For instance, MWCNTs decorated with Pt nanoparticles showed excellent features in the sensor for monitoring hydrogen peroxide concentrations [17]. Platinum nanoparticles are more often employed and supported onto carriers rather than being used separately. This is because they can easily agglomerate or deactivate while supporting them onto carriers enhances their stability and durability [26]. Also, in the hybrid version, the surface area available for electrochemical reactions is much higher; the dispersion of nanoparticles is uniform and supports MWCNTs, which can facilitate the adsorption of reactant molecules onto the electrode’s surface. MWCNTs can alter the electronic structure and chemical environment of Pt nanoparticles, thereby allowing for the tuning of their activity and selectivity, which is the case in numerous reports dealing with this matter [27].

In this paper, we synthesized Pt-doped MWCNTs, which were used for the fabrication of two ionophores, (a) Pt@MWCNT-DHBI and (b) Pt@MWCNT-DBS ionophores. These ionophores were used, together with a new Elvaloy 742 plasticizer, to fabricate two sensing membrane nanocomposites for potentiometric applications in surfactant analysis. Platinum was selected since it offers high conductivity and chemical stability. The sensors were characterized, and the selected sensor was successfully tested on technical grade and commercial product samples. The presented sensing platform featured advantages like fast and low-cost preparation, high stability and sensitivity, and a wide linear dynamic range. Also, this electrochemical setup for the effective determination of surfactant concentrations can be employed as a part of portable devices for on-site use. In addition to the technical improvement of the prepared composite material, there is a significant saving in the costs of applying platinum in such a highly dispersed version instead of pure metal.

## 2. Materials and Methods

### 2.1. Chemicals

Potentiometric measurements were carried out for the following anionic surfactants: sodium salts SDS—dodecylsulfate and DBS—dodecylbenzenesulfonate (Fluka, Buchs, Switzerland), cationic surfactants Hyamine 1622—benzethonium chloride, CPC—cetylpyridinium chloride, and CTAB—hexadecyltrimethylammonium bromide (Merck, Munich, Germany). The pH was adjusted using corresponding amounts of NaOH and HCl (all from Kemika, Zagreb, Croatia). HNO_3_, H_2_SO_4_, H_2_PtCl_6_, and NaBH_4_ were used for the doping process (all from Kemika, Zagreb, Croatia).

For the preparation of the sensing membrane nanocomposite, a high molecular weight PVC (Sigma Aldrich, Taufkirchen, Germany) and plasticizer Elvaloy 742 (DuPont, Wilmington, DE, USA) were used.

For interference measurements, the most common interfering cations were used, such as ${NH}_{4}^{+}$, Ca^2+^, Na^+^, Mg^2+^, and $C_{8}H_{20}N^{+}$ (Merck, Munich, Germany).

### 2.2. Pt-Doped MWCNT Synthesis

For the sake of doping with platinum, 150 mg of the activated MWCNTs were suspended in the mixture containing 10 mL of ethanol and 10 mL of deionized water and sonicated for 10 min. Then, 0.5 mL of the aqueous solution of H_2_PtCl_6_ (160 g/L) was introduced and sonicated for another 5 min. The reduction in Pt ions to their elemental state was performed by the addition of 60 mg of NaBH_4_ previously dissolved in 20 mL of deionized water. Platinum doping effects were confirmed by our previous study [28]. After 24 h, probes were filtrated on the Buchner funnel with copious amounts of deionized water, dried, and Pt-doped acid-activated MWCNTs were produced according to the following procedure. For the purpose of chemical activation, 500 mg of MWCNTs (Merck, Munich, Germany) was heated at 50 °C with 20 mL of the mixture of concentrated acids (HNO_3_:H_2_SO_4_ = 1:3) under reflux for 210 min. Activated MWCNTs were rinsed at the Buchner funnel with at least 1 L of deionized water, then dried for a couple of hours, homogenized, and kept in a glass cuvette for further procedure. This step is crucial for the introduction of hydroxyl functional groups in the structure of MWCNT.

### 2.3. Pt@MWCNT-DHBI Ionophore Production

The 1,3-dihexadecyl-1H-benzo[d]imidazol-3-ium (DHBI) cationic surfactant synthesis was described in our previous paper [29]. The synthesis of the ionophore based on carbon nanotubes’ complex with a cationic surfactant was presented for the first time recently [15]. This ionophore was used for surfactant selective electrochemical sensor fabrication. In this work, we performed a modification of this protocol to enhance the Pt@MWCNT and cationic surfactant interaction and the inclusion of complex formation. In total, 5 mL of hexane was added to 0.05 g of MWCNT and stirred for 24 h. This was carried out to increase the hydrophobicity of the inner cavity of the Pt@MWCNT and enhance the inclusion of complex formation. Next, the Pt@MWCNTs were left to dry at room temperature. After this, 2 mL of the 5 × 10^−4^ M solution of DHBI-Br was added to the Pt-doped MWCNTs and stirred for 24 h. After filtration, the precipitate was washed with ultrapure water. Next, the precipitate dried at 80 °C. The prepared Pt@MWCNT-DHBI ionophore was ready for further processing.

### 2.4. Pt@MWCNT-DHBI Surfactant Sensor Fabrication

The sensor membrane nanocomposite was fabricated by mixing 3 mg of Pt@MWCNT-DHBI ionophore with 25 mL of THF (Fluka, Buchs, Switzerland) and 120 mg of high molecular weight PVC. Then, 180 mg of the Elvaloy 742 plasticizer was added and stirred by ultrasound for a few minutes. The cocktail was poured into a glass ring mold and left for 24 h to dry. The membrane was cut into small discs and one of these discs was mounted in the Phillips electrode body IS-561 (Glasblaeserei Moeller, Zurich, Switzerland) filled with 3 M of potassium chloride as an inner filling solution. The sensor was prepared and ready to use. The sensor was stored in deionized water between the measuring intervals.

### 2.5. Instrumentation

The distribution of Pt nanoparticles on the MWCNTs’ was examined using high-resolution transmission electron microscopy (HR-TEM) with the JEOL JEM-F200 instrument (JEOL, Tokyo, Japan).

The Pt@MWCNT and Pt@MWCNT-DHBI surfactant sensors were characterized by the Shimadzu IR solution 1.30 FTIR-8400 S infrared spectrophotometer (Shimadzu, Kyoto, Japan). For response, interference, and pH measurement, a Metrohm 794 Basic Titrino with the Metrohm 781 pH meter (Metrohm, Herisau, Switzerland) was used. For titrations, a Metrohm 808 Titrando (Metrohm, Herisau, Switzerland) was employed. The Ag/AgCl reference electrode was used in all measurements. The membrane resistance was measured by the UT15B Max True RMS Digital Multimeter (Uni-Trend Technology EU GmbH, Augsburg, Germany).

### 2.6. Response Procedure

Potentiometric response measurements were carried out by incrementally adding the selected cationic (CPC, CTAB) or anionic (SDS, DBS) surfactant to the fixed amount of deionized water. To reach the logarithmic activity range from approximately −2 to −7, the selected concentrations were 4 × 10^−3^ M and 4 × 10^−4^ M.

In order to investigate the interferences from selected anions, including chlorides, carbonates, nitrates, acetates, sulfates, borates, EDTA, dihydrogenphosphates, hydrogen carbonates, and hydrogen sulfates (all chemicals from Kemika, Zagreb, Croatia), tests were performed by incrementally adding SDS in the 0.01 M interfering ion solution.

### 2.7. Titration Procedure

During potentiometric titrations, the dynamic equivalent point titration (DET) mode with a signal drift of 5 mV/min was used. At higher concentrations, the waiting time was 15 s. To reach the signal stabilization at lower concentrations, a 30 s waiting time was used.

For titrations of the technical grade cationic surfactant Hyamine 1622 (4 × 10^−3^ M), a corresponding concentration of SDS was used (4 × 10^−3^ M).

For titrations of commercial products containing anionic surfactants, variable concentrations of CPC were used.

After each measurement, the surfactant sensor was washed with deionized water.

## 3. Results

### 3.1. TEM Characteristics of Pt@MWCNT

**Figure 1:**
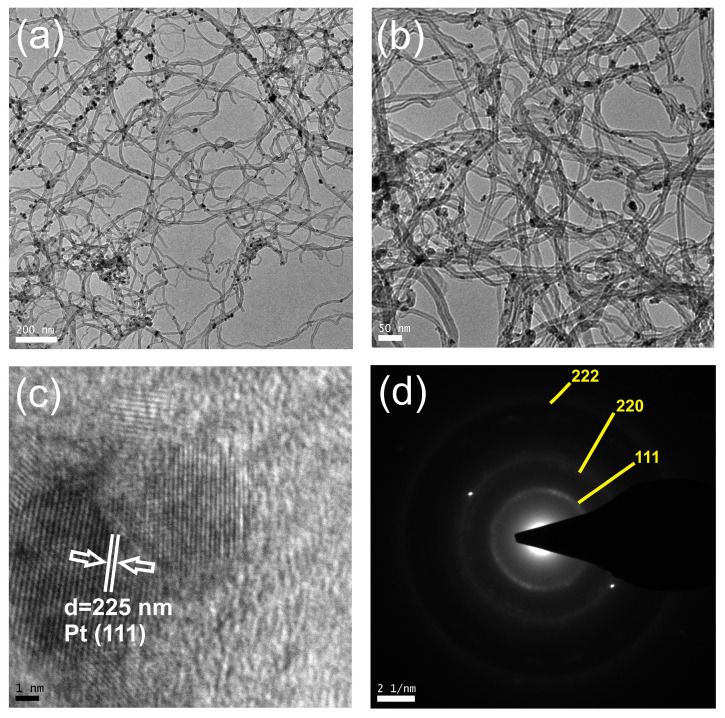
TEM image of Pt-doped MWCNTs: (**a**,**b**) at different magnifications, (**c**) HRTEM image showing a cluster of Pt nanoparticles and (**d**) Selected Area Electron Diffraction (SAED) patterns.

The representative structure of the Pt@MWCNTs analyzed by Transition Electron Microscopy is illustrated in Figure 1. An effective dispersion and deposition of Pt nanoparticles onto the CNT support can be seen in Figure 1a without evidence of filling the interior of the MWCNT with Pt. A higher magnification, as obtained on the TEM image in Figure 1b, reveals that Pt nanoparticles tend to agglomerate, forming clusters with an average diameter of approximately 10 nm. It is noteworthy that Pt nanoparticles firmly adhere to the surface of nanotubes, as no detached particles were observed. In the HRTEM image depicted in Figure 1c, a cluster of nanoparticles is visible, with a measured D-space of around 0.225 nm, corresponding to the spacing of (111) planes. The Selected Area Electron Diffraction (SAED) patterns of the Pt nanoparticles (Figure 1d) are indexed to three primary planes, (111), (220), and (222), indicating the random orientation of the Pt nanoparticles.

### 3.2. IR Characteristics of Pt@MWCNT, Pt@MWCNT-DHBI and Pt@MWCNT-DBS

After the Pt@MWCNT, Pt@MWCNT-DHBI, and Pt@MWCNT-DBS synthesis, the compounds were characterized by FT-IR spectrometer to observe the nanocomposites in the IR spectra and check the stability of the ionophore nanocomposite.

**Figure 2:**
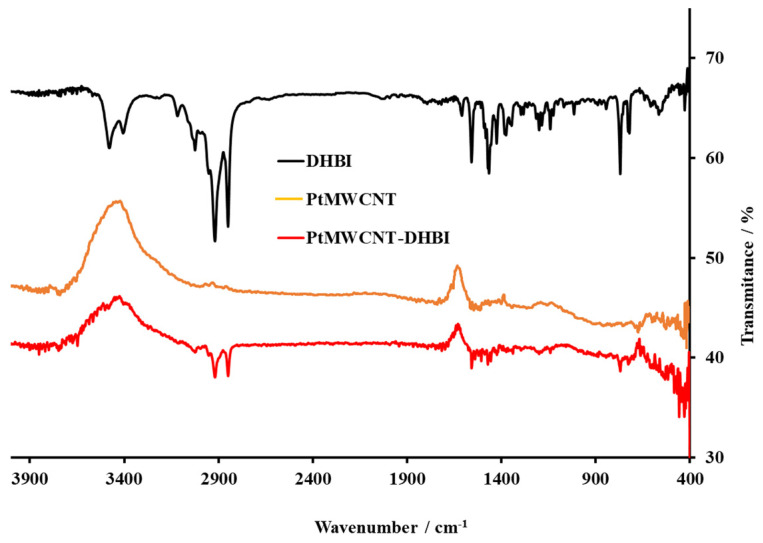
The FT-IR spectra of the DHBI cationic surfactants, the Pt@MWCNT nanomaterial, and the complex of Pt@MWCNT-DHBI in KBr. The DHBI values were adapted to fit the other spectra.

Figure 2 shows the IR spectra of the Pt-doped MWCNTs (PtMWCNT), pure DHBI cationic surfactant, and a doped nanomaterial after the formation of a complex with DHBI (PtMWCNT-DHBI). The spectra of acid-functionalized MWCNT dopped with platinum show several distinctive peaks. The intensive band at 3450 cm^−1^ originates from the stretching vibrations of isolated surface -OH groups, -OH in carboxyl groups, and/or sorbed water in the sample. The weakly expressed stretching peak from the C-O bond can be barely noticed around 1350 cm^−1^. A slim peak near 2950 cm^−1^ comes from the asymmetric stretching of methyl or methylene groups, usually located at the defect sites on the sidewall surface. The peak at 1560 cm^−1^ is related to the carboxylate anion stretch mode, and this peak is not seen on pristine MWCNT. The characteristic fingerprint regions of the DHBI cationic surfactant, 3025 cm^−1^ and 2960 to 2746 cm^−1^ (ν CH), and aromatic benzene ring shown by signals 1600 and 1465 cm^−1^ (ν CC aromatic stretching) can be also observed at the IR spectra of the PtMWCNT-DHBI complex. In this way, the synthesis was successful, and the complex was formed and ready to use for membrane fabrication and PtMWCNT-DHBI sensor characterization.

**Figure 3:**
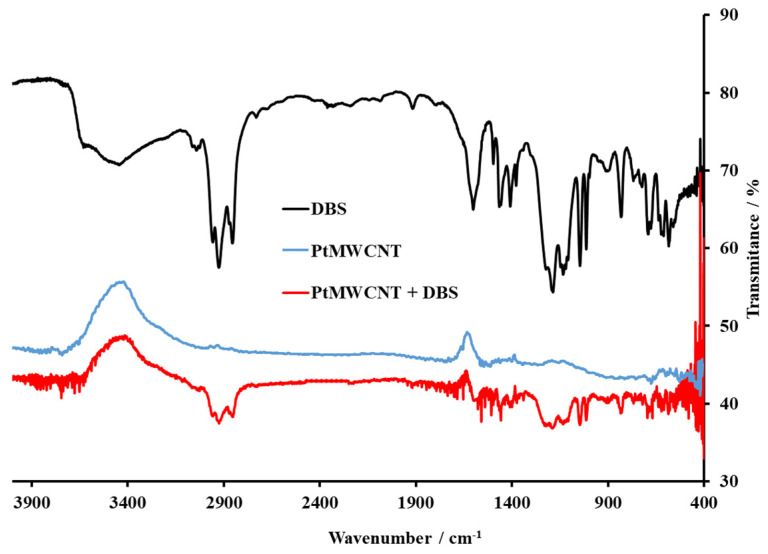
The FT-IR spectra of the DBS anionic surfactants, the Pt@MWCNT nanomaterial, and the Pt@MWCNT-DBS complex in KBr. The DBS values were adapted to fit the other spectra.

Figure 3 shows the IR spectra of the Pt-doped MWCNTs (PtMWCNTs), pure anionic surfactant DBS, and a doped nanomaterial after the formation of a complex with DBS (PtMWCNT-DBS). As far as the DBS spectrum is concerned, the broadband in the region 3200–3600 cm^−1^ is attributed to O-H bond stretching and indicates the presence of humidity in the solid surfactant sample. The band around 3100 cm^−1^ is related to C-H aromatic stretching, while the bands slightly below 3000 cm^−1^ are attributed to the vibration of the axial deformation of C-H for the CH_2_ group of the surfactant tail. In this region, the band may be located from the aromatic stretching vibrations of C-H. The rest of the DBS surfactant spectrum can be considered through the characteristic fingerprint regions, including the widened band around 3038 cm^−1^, peaks from 2875 to 3005 cm^−1^, from 1533 to 1690 cm^−1^, peaks at 1405 cm^−1^, 1457 cm^−1^ and from 1060 to 1273 cm^−1^. These regions can generally be attributed to the OH groups, C-H_2_ groups, C-O-C groups, and to the aromatic structure of DBS. The characteristic fingerprint regions of two major spectral regions corresponding to the hydrophobic tail, 3005 to 2875 cm^−1^ (ν CH), and the hydrophilic sulfonate region from 1270 to 900 cm^−1^ (ν_as_ S=O (board, very strong) and νs S=O (sharp, very strong)) of DBS can be also observed at the IR spectra of the PtMWCNT-DBS complex. In this way, the synthesis was successful, and the complex was formed and ready to use for membrane fabrication and PtMWCNT-DBS sensor characterization.

### 3.3. Response Characteristics of Fabricated Surfactant Sensors

The response of the prepared Pt@MWCNT-DHBI surfactant sensor was investigated in deionized water with the incremental addition of cationic surfactants CTAB and CPC, respectively. The increment volumes were calculated according to the achieved concentration of the corresponding cationic surfactant to cover a wide concentration range. The response mechanism of the proposed Pt@MWCNT-DHBI surfactant sensor to cationic surfactants can be described as follows:

(1) $$E=E^{o}+S\;log\;a_{cat.surf.}$$

The proposed equation is a modified Nernst equation, in which E represents the electromotive force, $E^{o}$ is a constant potential term, and $a_{cat.surf.}$ is the activity of the corresponding cationic surfactant.

The response mechanism of the proposed Pt@MWCNT-DHBI surfactant sensor to anionic surfactants can be described as follows:

(2) $$E=E^{o}-S\;log\;a_{an.surf.}$$

The proposed equation is a modified Nernst equation, in which E represents the electromotive force, $E^{o}$ is a constant potential term, and $a_{an.surf.}$ is the activity of the corresponding anionic surfactant.

Response characteristics were tested in deionized water for both fabricated surfactant sensors, Pt@MWCNT-DHBI and Pt@MWCNT-DBS. Both sensors were tested in the presence of anionic surfactants SDS and DBS and cationic surfactants CPC and CTAB, respectively.

**Figure 4:**
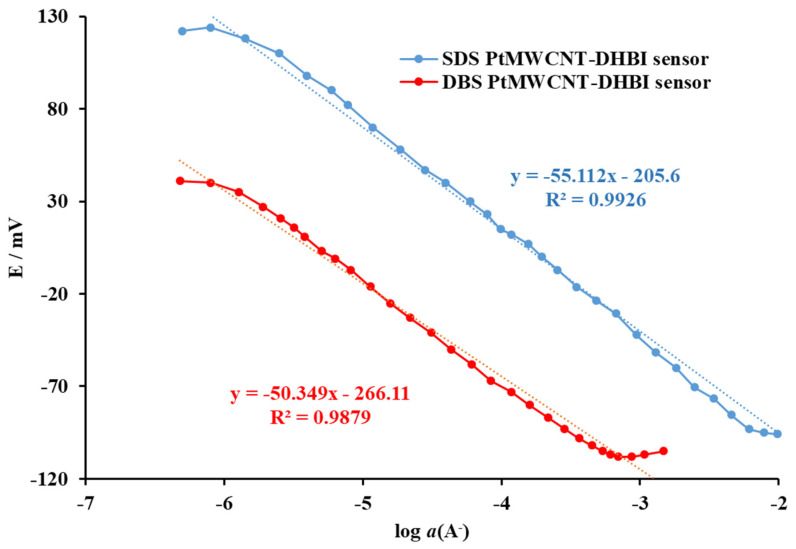
The electromotive force of the Pt@MWCNT-DHBI surfactant sensor as a function of the incremental addition of SDS and DBS anionic surfactants (A^−^) in deionized water at 25 °C. The curves were displaced for clarity. The trend lines were added for orientation.

The response characteristics of the proposed surfactant sensor Pt@MWCNT-DHBI for anionic surfactants SDS and DBS are shown in Figure 4. The response calibration curves were linear over a wide concentration range with a distinct inflexion for both anionic surfactants SDS and DBS.

The statistical evaluation of the response of the Pt@MWCNT-DHBI surfactant sensor to SDS and DBS is presented in Table 1. The Pt@MWCNT-DHBI surfactant sensor showed a Nernstian response to SDS (59.1 mV/decade of activity) and a slightly sub-Nernstian response to DBS (57.5 mV/decade of activity). The linear response regions for both anionic surfactants were broad, up to ≈2 × 10^−6^ M. This was important since it could allow the quantification of anionic surfactants over a broad concentration range, including higher but also lower anionic surfactant concentrations. Additionally, the critical micellar concentration (CMC) for both anionic surfactants was in agreement with the literature [30]. Using the proposed sensor, the CMCs for anionic surfactants could be detected.

**Figure 5:**
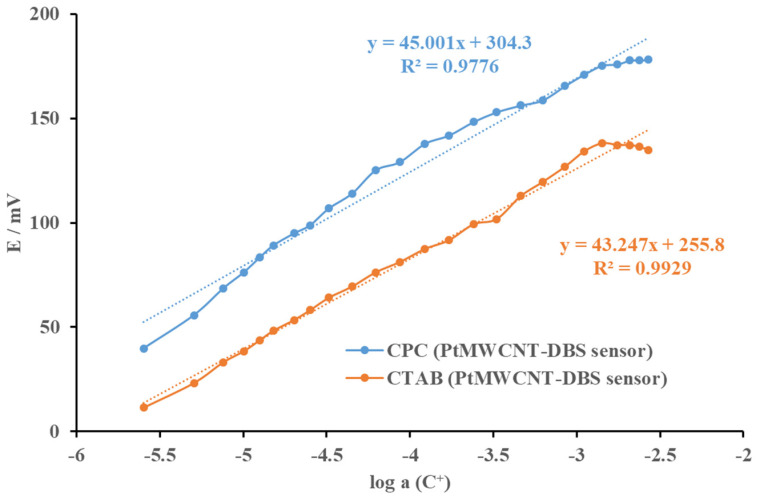
The electromotive force of the Pt@MWCNT-DHBI surfactant sensor as a function of the incremental addition of CPC and CTAB in deionized water at 25 °C. The curves were displaced for clarity. The trend lines were added for orientation.

The response characteristics of the proposed surfactant sensor Pt@MWCNT-DHBI were also tested for the cationic surfactants CPC and CTAB (Figure 5). The response calibration curves were linear over a wide concentration range with an inflexion for both cationic surfactants CTAB and CPS and CMC concentrations. The statistical evaluation of the response of the Pt@MWCNT-DHBI surfactant sensor to CPC and CTAB showed a sub-Nernstian response in the linear response region, with 49.2 mV/decade of activity for CPC and 44.7 mV/decade of activity for CTAB. The linear response region for CTAB was broader compared to the CPC. Even though the proposed Pt@MWCNT-DHBI surfactant sensor showed good response characteristics, the sub-Nernstian response for both surfactants reduced the sensitivity in cationic surfactant quantification.

**Figure 6:**
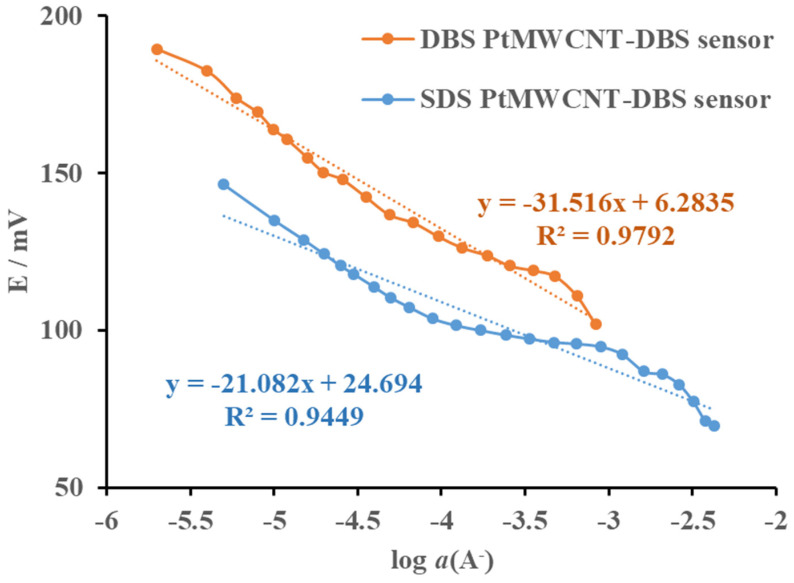
The electromotive force of the Pt@MWCNT-DBS surfactant sensor as a function of the incremental addition of CPC and CTAB cationic surfactants (C^+^) in deionized water at 25 °C. The curves were displaced for clarity. The trend lines were added for orientation.

The proposed Pt@MWCNT-DBS surfactant sensor was tested in response to the anionic surfactants SDS and DBS (Figure 6). The sensor exhibited a sub-Nernstian response for both anionic surfactants. The useful linear response region for DBS had a slope of 32.1 mV/decade of activity, while the linear response region for SDS could be observed starting from 1 × 10^−4^ M, with a slope of 33.8 mV/decade of activity. The Pt@MWCNT-DBS surfactant sensor exhibited limited potential usage for anionic surfactant quantification.

**Figure 7:**
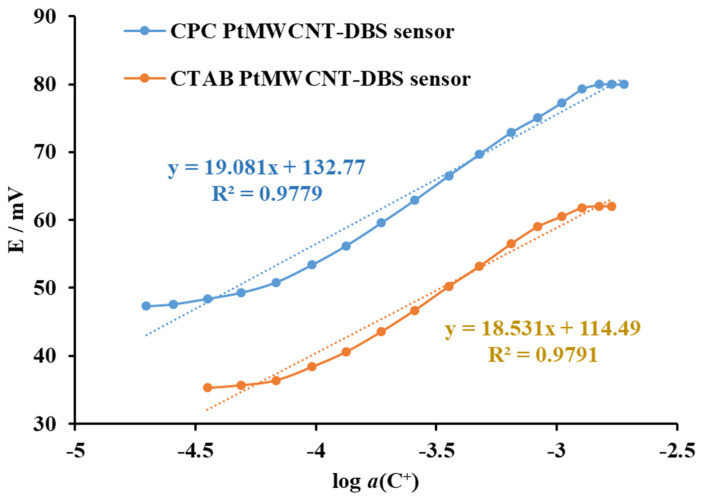
The electromotive force of the Pt@MWCNT-DBS surfactant sensor as a function of the incremental addition of CPC and CTAB cationic surfactants (C^+^) in deionized water at 25 °C. The curves were displaced for clarity. The trend lines were added for orientation.

The Pt@MWCNT-DBS surfactant sensor was tested in response to the cationic surfactants CPC and CTAB (Figure 7). The sensor exhibited a sub-Nernstian response for both anionic surfactants. The useful linear response region (1 × 10^−4^ to 1 × 10^−3^ M) for CPC had a slope of 23.7 mV/decade of activity, while the linear response region for CTAB (1.6 × 10^−4^ to 1 × 10^−3^ M) had a slope of 22.9 mV/decade of activity. The Pt@MWCNT-DBS surfactant sensor exhibited potential usage for the quantification of cationic surfactants in a very narrow region.

When comparing the two sensors, Pt@MWCNT-DHBI sensors showed superior characteristics compared to Pt@MWCNT-DBS sensors. The sensitivity was two times higher for the same investigated regions, and the useful linear regions were broad, allowing the potential application of the sensor to quantify both anionic and cationic surfactants in water and commercial products. The sensor was further characterized by interferences.

### 3.4. The Interference Study of Pt@MWCNT-DHBI Sensor

The interference study was performed to observe the response of the Pt@MWCNT-DHBI surfactant sensor to selected interfering anions (Table 2). The solution of the selected interfering cation was used for the incremental addition of SDS. The fixed interference method (FIN) proposed by IUPAC was used to calculate the interfering influence. The calculated ${logK}_{Surf}^{Pot}$ for selected anions was in the range from −3.2 to 4.7. From the $K_{Surf}^{Pot}$ values, it can be concluded that the usual anions had a minor influence on the response characteristics of the surfactant sensor Pt@MWCNT-DHBI.

A low influence of potentially interfering anions was also achieved using the membrane composition proposed recently [15]. The selected plasticizer, Elvaloy 742, is a terpolymer of ethylene, vinyl acetate, and carbon monoxide; it contributed to the stability and response of the sensing membrane because it is a permanent, non-migrating plasticizer (with a molecular weight of about 250,000) that provides toughness and flexibility that are locked into the PVC. In essence, membranes could be considered compatible polyblends rather than flexible PVC [31]. The measured sensor drift was ≈6 mV per hour. The combination of the proposed sensor membrane composition with the selected plasticizer and Pt-doped MWCNT-based ionophore allowed the high lipophilicity of the membrane, lower noise, a higher surface area, higher stability, and better charge transfer. The use of a Pt-MWCNTs-based ionophore and a new plasticizer had a positive influence on leaching since the ionophore is more bound inside the sensor membrane matrix.

Before each use, the sensor was conditioned in a solution of the cationic surfactant CTAB (4 × 10^−^^3^ M). If the sensor was not used for several weeks, it was stored dry. Before each new series of measurements, the sensor was conditioned using the same procedure. In this way, it was possible to maintain the reproducibility and stability of the electrode. In addition, the use of Pt-doped MWCNTs extended the lifetime by more than six months.

### 3.5. Titration of Technical Grade Cationic Surfactant with Pt@MWCNT-DHBI Sensor

The titration of charged surfactants, both anionic ($A^{-}$) and cationic ($C^{+}$), is described by the formation of the low-solubility ion pair (CA):

(3) $$A^{-}+C^{+}\Leftrightarrow CA$$

**Figure 8:**
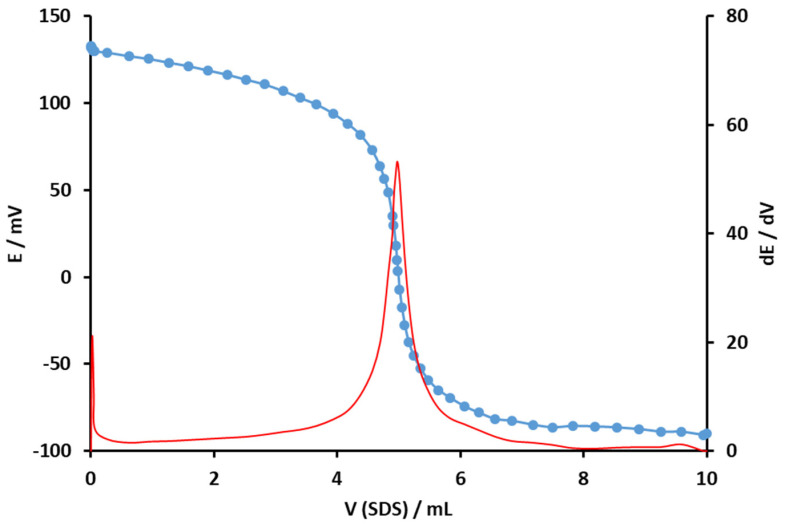
Potentiometric titration (blue) and 1st derivative (red) of a technical grade cationic surfactant Hyamine 1622 titration with anionic surfactant SDS (4 × 10^−3^ M) and the Pt@MWCNT-DHBI surfactant sensor as an end-point indicator.

Finally, the proposed surfactant sensor Pt@MWCNT-DHBI was tested as an end-point indicator in titrations with the cationic surfactant Hyamine 1622. The titration curve was sigmoidal and smooth, with a total signal change of 222.7 ± 9.2 mV (Figure 8). The inflexion point was well-defined and exhibited a sharp signal drop. The first derivative curve showed a sharp peak at the end-point, and the value was well-defined.

The surfactant sensor Pt@MWCNT-DHBI was also used as an end-point indicator for potentiometric titrations of solutions with defined amounts of cationic surfactant added, i.e., by the standard addition method. The added amounts were 50 and 10 µmol of the selected cationic surfactants CTAB and Hyamnie 1622, respectively (Table 3). The results exhibited recoveries ranging from 98.2 to 99.2%.

### 3.6. Titration of Commercial Products with Pt@MWCNT-DHBI Sensor

Six samples of detergents for various applications were purchased in local stores. All of them had declared the content of anionic surfactants. The samples were tested, and the amount of anionic surfactant was calculated based on the end-point potentiometric titration results. As a titrant, a CPC in various concentrations was used. The results presented in Table 4 show the amounts of anionic surfactants measured by the Pt@MWCNT-DHBI surfactant sensor and compared with the ISE surfactant sensor previously published by our group. The results were also compared with the classical two-phase titration method. The results showed good agreement with other methods.

The Pt@MWCNT-DHBI surfactant sensor was successfully exploited for several months on a daily basis. After checking the response signals from the first days of use with the data from six months of use, there was no significant signal change. This lack of leaching and extended lifetime could be explained by the formation of a strong complex between the acid-activated and Pt-doped MWCNTs with the positively charged DHBI surfactant and the use of a new plasticizer.

## 4. Conclusions

Two different ionophores using Pt-doped acid-activated MWCNTs in combination with the cationic surfactant DHBI (Pt@MWCNT-DHBI ionophore) and anionic surfactant DBS (Pt@MWCNT-DBS ionophore) were successfully produced and characterized. The use of the new plasticizer Elvaloy 742 in combination with Pt-doped MWCNT-based ionophores was successfully employed in sensing membrane nanocomposite synthesis.

The potentiometric response of both sensors, Pt@MWCNT-DHBI and Pt@MWCNT, to anionic surfactants (SDS and DBS) and cationic surfactants (CPC and CTAB) showed that the Pt@MWCNT-DHBI surfactant sensor revealed superior response properties to SDS (59.1 mV/decade of activity) and to DBS (57.5 mV/decade of activity), with linear response regions for both anionic surfactants (up to ≈2 × 10^−6^ M).

The Pt@MWCNT-DHBI surfactant sensor was selected for further analysis. The interference tests showed the low interference influence of common anions and SDS response measurements. The sensor was successfully employed in the potentiometric titration of a technical grade cationic surfactant with recoveries ranging from 98.2 to 99.9%. The sensor was successfully used for the quantification of anionic surfactants in six samples of different commercial detergents. The results were compared with the ISE surfactant sensor and the classical two-phase titration method and showed good agreement.

The use of a Pt@MWCNT-based ionophore and a new plasticizer prevented the leaching, which resulted in an extended lifetime. For these reasons, the Pt@MWCNT-DHBI surfactant sensor is a promising tool for surfactant quantification in product formulations but also in other aqueous samples, like wastewater.

## Figures and Tables

**Table 1:** Statistical evaluation of response characteristics of the Pt@MWCNT-DHBI surfactant sensor for anionic surfactants SDS and DBS.

Parameters	SDS	DBS
Slope (mV/decade)	59.1 ± 0.2	57.5 ± 0.3
Correlation coefficient (R^2^)	0.9993	0.9898
Intercept (mV)	223 ± 2	289 ± 3
Useful linear concentration range (M)	2.5^−6^ to 6.3 × 10^−3^	1.8 × 10^−6^ to 4.5 × 10^−4^

**Table 2:** Calculated logarithm of selectivity coefficient for most common inorganic and organic anions (0.01 M) measured by the Pt@MWCNT-DHBI surfactant sensor and adding SDS.

Interfering Anions	${logK}_{{An}_{i.}^{-}}^{pot}$
Chloride	−3.2
Carbonate	−4.8
Nitrate	−3.3
Acetate	−4.5
Sulfate	−3.9
Borate	−4.4
EDTA	−4.7
Dihydrogenphosphate	−3.9
Hydrogen carbonate	−3.2
Xylensulfonate	−3.6
Fluoride	−4.3
Bromide	−4.5
Hydrogen sulfate	−3.9

**Table 3:** Results for potentiometric titration of technical grade cationic surfactants CTAB and Hyamine 1622 by the standard addition method. The Pt@MWCNT-DHBI sensor was used as an end-point indicator, and SDS was used as a titrant (4 × 10^−3^ M), with mean values at ±95% confidence limits.

Cationic Surfactant	*n* (Added)/µmol	*n* (Found)/µmol	Recovery/%
CTAB	50	49.93 ± 0.06	99.8
	10	9.82 ± 0.04	98.2
Hyamine 1622	50	49.96 ± 0.03	99.9
	10	9.92 ± 0.07	99.2

**Table 4:** Comparison of anionic surfactant content in commercial products measured by potentiometric titration with the Pt@MWCNT-DHBI surfactant sensor, ISE surfactant sensor, and a two-phase titration.

Commercial Detergents		% Anionic Surfactant		
		Pt@MWCNT-DHBI	ISE Surfactant Sensor *	Two-Phase Titration **
Powder	sample 1	6.3 ± 0.1	6.3	6.15
	sample 2	6.1 ± 0.1	6.0	6.09
Liquid gel	sample 3	2.1 ± 0.1	2.1	2.01
	sample 4	2.1 ± 0.1	2.2	2.09
Handwashing	sample 5	13.2 ± 0.1	13.2	13.5
	sample 6	15.1 ± 0.1	15.1	15.3

* surfactant sensor presented in [32]; ** referent method presented in [2].

## Data Availability

Data are contained within the article.
